# Effects of soil and topographic factors on vegetation restoration in opencast coal mine dumps located in a loess area

**DOI:** 10.1038/srep22058

**Published:** 2016-02-26

**Authors:** Jinman Wang, Hongdan Wang, Yingui Cao, Zhongke Bai, Qian Qin

**Affiliations:** 1 College of Land Science and Technology, China University of Geosciences, 29 Xueyuanlu, Haidian District, 100083 Beijing, People’s Republic of China; 2 Key Laboratory of Land Consolidation and Rehabilitation, Ministry of Land and Resources, 100035 Beijing, People’s Republic of China

## Abstract

Vegetation plays an important role in improving and restoring fragile ecological environments. In the Antaibao opencast coal mine, located in a loess area, the eco-environment has been substantially disturbed by mining activities, and the relationship between the vegetation and environmental factors is not very clear. Therefore, it is crucial to understand the effects of soil and topographic factors on vegetation restoration to improve the fragile ecosystems of damaged land. An investigation of the soil, topography and vegetation in 50 reclamation sample plots in Shanxi Pingshuo Antaibao opencast coal mine dumps was performed. Statistical analyses in this study included one-way ANOVA and significance testing using SPSS 20.0, and multivariate techniques of detrended correspondence analysis (DCA) and redundancy analysis (RDA) using CANOCO 4.5. The RDA revealed the environmental factors that affected vegetation restoration. Various vegetation and soil variables were significantly correlated. The available K and rock content were good explanatory variables, and they were positively correlated with tree volume. The effects of the soil factors on vegetation restoration were higher than those of the topographic factors.

China requires abundant natural resources for economic development, particularly resources obtained from coal mines. Currently, opencast coal mine production in China accounts for 12% of the total production. These opencast coal mines are primarily located in the vulnerable environments of northwestern China, such as Shanxi Province, Inner Mongolia, and Shaanxi Province^1^,^2^. With the incentives of rapid economic development, extensive mining areas are emerging in these regions. The Pingshuo Antaibao opencast coal mine began in 1985 in Shanxi Province and is located in the loess area of northwestern China. Substantial changes have occurred in the eco-environment, due to long-term and large-scale mining disturbances. The average annual destruction area of land was approximately 6.6 × 10^4^ hm^2^ in this mining area^3^. The mining activities caused changes in the soil structure and soil physicochemical properties, severely destroyed the vegetation, and formed a large dump area. The operation of heavy machinery also resulted in high soil compaction, large bulk density, deficient soil nutrients and major soil erosion. The dumps without revegetation had a total soil erosion of 15,060 t·km^−2^·a^−1^ or a 33% greater erosion rate than that of the original Loess Plateau landform, which has a typical soil erosion rate of 10,120 t·km^−2^·a^−1 ^4. Therefore, the restoration of the ecosystem has been very difficult in the mining area^5^.

As one of the important measures in governance of an ecological environment, vegetation restoration can make full use of the function of the soil-plant composite system, improve the local environment and promote a regional ecological balance^6^. At different scales, vegetation, soil and topographic factors are closely linked^7^. Therefore, it is very important to understand the effect of soil and topographic factors on vegetation restoration for land reclamation and ecological restoration in opencast coal mining areas.

Functional changes in vegetation are involved in the mechanism underlying the induction of some soil changes that favour an increase in the plant growth in mined areas^8^. Vegetation restoration affects the soil properties at different scales, and conversely, the soil characteristics and topographic features influence vegetation development^9^. Previous studies have demonstrated that vegetation growth is significantly positively correlated with the soil organic matter, total N, available P, and available K^10^,^11^. Vegetation restoration can also significantly improve the soil bulk density, soil water retention and soil porosity^12^,^13^. Conversely, the soil organic matter has a notable effect on vegetation and also has some effects on the other soil nutrients under conditions of vegetation growth and development^11^. However, a previous study has also found that vegetation restoration has little effect on the soil organic matter and available nitrogen in abandoned wasteland, and this effect depends on the restoration model of artificial vegetation^14^.

Furthermore, a better understanding of the influence of topographic gradients on vegetation restoration has long been a goal of vegetation managers and ecologists^15^. A study conducted to study the early stage of the relationship between vegetation and topographic factors has found that landscape variations in vegetation are strongly related to topographic factors^16^,^17^, and shrub land vegetation patterns frequently show a strong correlation with the slope position in Nevada, USA^18^. The tree density is negatively related to the elevation, slope, and slope aspect in the remnant moist Afromontane forest of Wondo Genet, located in south central Ethiopia^19^. Previous studies in this area have indicated that the correlation between the diversity of species is essentially related to the slope position and altitude.

Previous studies on the relationship of vegetation, soil and topographic factors have always been conducted using traditional multivariate analysis, but the results may be subjective due to the high number of environment variables and the great fluctuation in topography^20^. However, analysis based on CANOCO can avoid this disadvantage associated with data conversion and multivariate analysis, such as Canonical Correspondence Analysis (CCA) and Redundancy Analysis (RDA)^21^.

In areas undisturbed by opencast coal mining, the effects of soil properties and topographic conditions on vegetation growth have been reported; however, studies in opencast coal mining area are rare, especially in using CCA or RDA method. Thus, the objective of this study was to study the effect of soil and topographic factors on vegetation restoration using CCA and RDA and to determine the relationship of vegetation, soil and topographic factors in an opencast coal mine area.

## Results

### Descriptive statistical analysis of the soil data

Minimum and maximum values were used as estimates of the variability in soil properties. The results of the descriptive statistical analysis showed that a large difference between the minimum and maximum values of the soil factors, particularly the soil organic matter, and the values of these factors followed a normal distribution (Table 1). The mean and median values were used as primary estimates of the central tendency. In addition, the mean and median values were mostly similar, with the majority of the median values being smaller than the mean values for the soil properties. This finding indicated that the outliers did not dominate the measures of central tendency. The CV values were also used as estimates of the variability in the soil properties. The soil bulk density and soil water content showed low CV values (<15%), and the soil total porosity, silt content, sand content and available K had moderate CV values (15–35%). The soil organic matter, total N, available P and rock content had high CV values (>35%), and the clay content had the highest CV (109%). Overall, the descriptive statistics showed high soil variability in the study area.

### One-way ANOVA analysis results

To analyse the degree of effects of soil and topographic factors on vegetation changes, a one-way ANOVA and significance testing were performed using SPSS 20.0. The results indicated that the effects of different soil and topographic factors on vegetation changes presented marked differences (Table 2). The effects of available K on tree volume were highly significant (*P* < 0.01), and the clay content had significant effects on tree volume (*P* < 0.05). However, the contribution of other soil factors and topographic factors was not significant (*P* > 0.05). This finding indicated that soil factors were the main factors that affected the restoration and reconstruction of vegetation in an opencast coal mine dump located in a loess area.

### DCA and RDA results

The ordination analysis showed the environmental gradient in the area (Table 3). The gradient lengths, as retrieved by DCA, were 1.706, 0.763, 0.853, and 0.000 from the first to the fourth axis, respectively. The results of the DCA analysis indicated that the gradient length of the first axis was less than 3 SD. Therefore, the linear model with RDA was considered to be the appropriate ordination method for direct gradient analysis.

**Figure 1:**
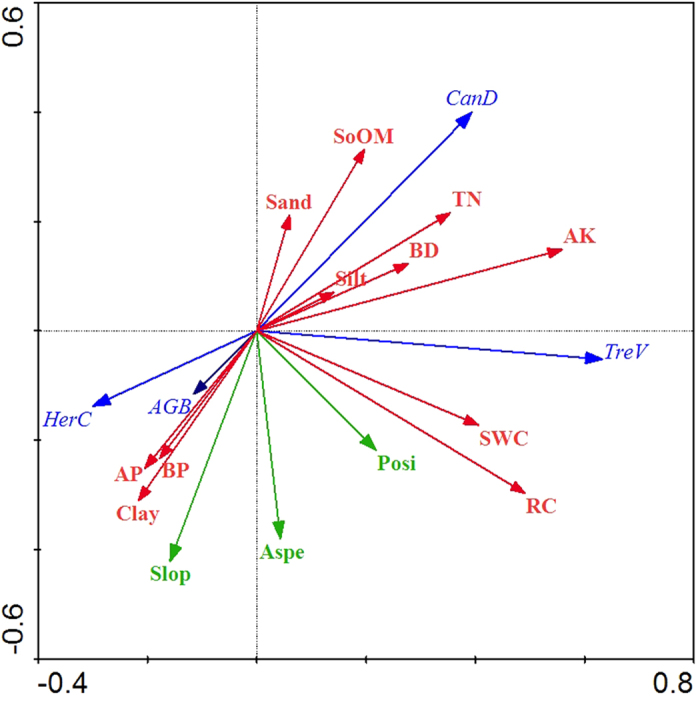
Ordination diagram showing the result of the RDA analysis of vegetation, soil, and topographic variables. Abbreviations of vegetation, soil, and topographic variables: TreV, tree volume; HerC, herb coverage; AGB, above-ground biomass; CanD, canopy density; Slop, slope; Posi, slope position; Aspe, slope aspect; SWC, soil water content; BD, soil bulk density; BP, soil total porosity; RC, rock content; SoOM, soil organic matter; TN, total N; AP, available P; and AK, available K.

RDA can independently retain the contribution rate of each soil and topographic variable on vegetation changes and it can evaluate the correlation between a variable and multivariable data based on statistical theory by identifying the variable that serves as the best predictor of the distribution of vegetation. Figure 1 presents the RDA ordination diagram. In the ordination diagram, a red arrow represents the soil factors, a green arrow represents the topographic factors, and a blue arrow represents the vegetation variables. The cosine values between the environmental variables (soil and topographic factors) and vegetation variables represent the correlation between corresponding variables. A positive cosine value indicates a positive correlation between the respective variables, whereas a negative cosine value indicates a negative correlation. The directions of the arrows indicate the direction of the change in the soil and topographic variables, and the lengths of the arrows indicate the extent to which this factor represents the vegetation data. The effects of the soil factors on vegetation restoration were higher than those of the topographic factors. The available K and rock content were the best explanatory variables for soil factors, followed by soil water content, soil organic matter, clay content, total N and available P. The slope and slope aspect exhibited a high explanatory power of the vegetation changes for topographic factors. The explanatory power of the other factors was relatively low.

There was a strong correlation between vegetation and environmental factors (soil and topography), with vegetation–environment correlations of 0.636 on the first axis and of 0.492 on the second axis. The cumulative percentage variance of the vegetation occurrence data explained by the first four axes of the RDA was 37.1%. The cumulative percentage variance of the vegetation–environment relationship on the first axis was 89.7%, whereas that on second axis was 6.6%; in other words, the first and second axes explained 96.3% of the relationship between the vegetation and the environment. This finding indicated that the vegetation and environment axes were highly correlated with the studied set of variables. The Monte Carlo permutation test indicated that vegetation restoration was correlated with the tested environmental factors (*P* < 0.05).

### Correlation among the explanatory variables

Figure 1 illustrates the relationships among the probabilities for vegetation and environment variables (soil and topography), and the first ordination axis represents the effects of the soil properties on vegetation restoration. The available K and rock content were the best explanatory variables compared with the other environmental variables, and they were positively correlated with tree volume. The soil water content was also positively correlated with tree volume. The soil organic matter and total N were positively correlated with canopy density and were negatively correlated with above-ground biomass and herb coverage, and high correlations were observed between these variables. With high correlations, the available P and soil total porosity were positively correlated with above-ground biomass and herb coverage and was negatively correlated with canopy density. The clay content was positively correlated with the herb coverage and above-ground biomass and was negatively correlated with canopy density. The silt content and sand content were positively correlated with canopy density and were negatively correlated with herb coverage and above-ground biomass. Although the explanatory power for the vegetation of silt content and sand content was relatively low, the soil texture also presented a high explanatory power for vegetation changes. The second ordination axis represents the effects of the topographic variables on vegetation. The slope and slope aspect were positively correlated with above-ground coverage and herb coverage, and were negatively correlated with canopy density. This finding showed that the effects of topographic factors on vegetation restoration in an opencast coal mine dump located in a loess area were not obvious, but the relationship between the soil factors and the vegetation data was clear.

The correlation between soil factors and topographic factors obtained through the RDA is shown in Table 4. The available P was negatively correlated with slope (−0.213). The total N was positively correlated with slope position (0.379) and slope aspect (0.251). The soil water content was positively correlated with the slope. THE rock content was positively correlated with slope and slope position. The analysis of the soil texture variables revealed that the silt content showed a significant negative correlation with the slope (−0.210). In contrast, the sand content presented a significant positive correlation with the slope and slope position. The clay content and sand content did not have any clear correlation with the topographic factors. The correlation between other soil factors and topographic factors was also not obvious (Table 4). The results indicated that variations in the slope, slope position, and slope aspect affected the soil variables.

The correlation analysis between the soil variables is shown in Table 4. The soil bulk density and soil total porosity showed a significant negative correlation (−0.961). The clay content showed a clear negative correlation with total N (−0.509) and soil organic matter (−0.468), and it showed a positive correlation with available P (0.311). The silt content showed a positive correlation with the soil water content (0.268) and a significant negative correlation with clay (−0.422). The sand content showed a significant negative correlation with silt content (−0.576) and clay content (−0.498). The sand content also showed a significant positive correlation with total N (0.495) and a positive correlation with soil organic matter (0.354). These changes were consistent with previous findings. The rock content and soil organic matter showed positive correlations with the total N (0.326). The total N was significantly and positively correlated with the soil organic matter (0.875). Furthermore, total N was positively correlated with available K (0.304). The soil organic matter was negatively correlated with the clay content (−0.468) and was positively correlated with the sand content (0.354). The available K was positively correlated with soil bulk density (0.207). The results indicated that the soil texture, among all of the soil physical properties, presented a clear predictive role of the soil nutrient content, and a clear correlation was detected among the soil nutrient indices. In addition, there was a clear correlation between the soil nutrients.

## Discussion

### Interrelationship between soil nutrients and vegetation restoration

Vegetation restoration plays a clear, predictive role in determining the soil nutrient content^22^, and soil variables, such as the available K and total N, have a significant influence on vegetation growth and development^23^,^24^,^25^. The present study indicated that the available K was the variable with the highest explanatory power compared with the other soil nutrient variables, followed by total N and soil organic matter. The main vegetation types in the study area were locust and pine, which supplied a high amount of litter to the soil. In addition, as the most important species in the study area, locusts have a strong nitrogen fixation function^26^; thus, the available K and total N contents increased. Many studies have shown that the soil organic matter presents surface accumulation and has a significant influence on vegetation growth and development^20^,^27^. In addition, different vegetation restoration types may also increase the soil organic matter to different degrees^28^,^29^. Because a high amount of litter was introduced into the soil each year, its decomposition by microorganisms resulted in a greater amount of humus, and increases in the soil organic matter. This study also found that soil organic matter was positively correlated with canopy density and negatively correlated with above-ground biomass and herb coverage, and high correlations were observed between these variables. A possible reason for this finding was that vegetation could increase soil organic matter. In addition, the coal gangue content in the dumps may also have affected the content of the soil organic matter. During vegetation reconstruction processes, the coal gangue content may differ between different sampling plots. The coal gangue itself contains organic matter; therefore, the soil organic matter content increases with coal gangue weathering^30^.

### Effects of topography on vegetation restoration and soil properties

Micro-topographic factors (such as slope, slope aspect and slope position) exert a strong influence on plant community structure and species distribution^31^. The ordination results obtained in this study indicated that the slope was the major topographic factor that affected vegetation restoration, followed by slope aspect. The slope and slope aspect were positively correlated with above-ground biomass and herb coverage, and were negatively correlated with canopy density. However, the effect of topographic factors on vegetation were not significant compared with soil factors. A possible reason for this finding is that vegetation development is mainly attributed to topographic factors in areas lacking in soil factors, whereas when both soil factors and topographic factors are present, soil factors were assumed to be more important^20^,^21^. The main factors that affect the spatial distribution of plant diversity have been found to be the slope aspect and elevation in the Longjiao Mountain forest area of China, but when the change in elevation is less than 300 m, elevation has little influence on vegetation development^32^. In this study area, the difference between the maximum and minimum elevations in the sampling plots was 125.05 m (less than 300 m), which may not be sufficiently large to induce significant variations in water/thermal conditions and is also not sufficient to affect vegetation restoration^20^; therefore, we did not consider the elevation variable. Topographic factors affected not only vegetation but also soil. Topography can partly affect the accumulation and export of soil nutrients, thereby indirectly impacting plant distribution^33^. Some studies have indicated that changes in topography have clear influences on soil physical and chemical properties and soil water characteristics^34^,^35^. In this study, the available P was negatively correlated with slope, and the total N was positively correlated with slope position and slope aspect. The soil water content was positively correlated with the slope, and the rock content was positively correlated with slope and slope position. The results also showed that the silt content had a significant negative correlation with the slope. In contrast, the sand content presented a significant positive correlation with the slope and slope position. The clay content and sand content did not have any clear correlation with the topographic factors. These changes also illustrated the function of topography on soil properties.

### Ecological restoration measures in a mining dump in a loess area

The inter-association and mutual restriction between soil factors and vegetation restoration not only illustrated the role of soil factors during vegetation restoration, but also revealed the restorative and beneficial effects of vegetation restoration on soil. Therefore, to improve and restore the fragile ecological system in an opencast coal mine dump located in a loess area, the co-evolution of both vegetation and soil should be considered^20^. Effective management and conservation, particularly through approaches based on the emulation of natural disturbances, should take these differences into consideration by developing site-specific management guidelines for vegetation^36^. A key strategy for ecological restoration in an opencast coal mine dump located in a loess area involves improvements in soil conditions and increases in the area of artificial vegetation. In addition, the protection of artificial and natural vegetation under local environmental conditions should also be strengthened.

One study had found that a mixed model of locusts and pines has high survival rate, and the adaptability of locust and pine in the process of vegetation restoration is strong in the Antaibao opencast coal mine dump^37^. Therefore, the mixed model of locusts and pines can be selected to conduct revegetation for dumps. Moreover, this paper showed that the soil factors had a clear correlation with the vegetation variable, and the available K and rock content were the best explanatory variables for soil factors. The available K is usually deficient in mined soils^38^; moreover, the surface soil was very thick in the loess area. Therefore, surface soil covering and soil improvement are necessary to increase soil fertility and reduce the rock content. Soil nutrients also can be increased by the accumulation of plant litter after vegetation restoration^39^.

## Conclusions

Vegetation restoration was strongly correlated with soil factors, such as the available K, soil organic matter, total N, rock content, and soil bulk density. The available K and rock content were the variables that exerted the most important effects on vegetation restoration. Of the topographic factors, the slope and slope aspect had large influences on vegetation restoration. Soil particles, which represent one of the physical properties, played a clear and predictive role in determining the soil nutrients.

To improve and restore the fragile ecological system in an opencast coal mine dump located in a loess area, the co-evolution of both vegetation and soil should be considered, and the mixed model of locusts and pines can be selected to conduct vegetation restoration for dumps.

## Materials and Methods

### Study area

The study area was located at the Antaibao opencast coal mine, specifically at the geographical coordinates of 112°11′58″-113°30′ E, 39°23′-30°27′N in Pingshuo, Shanxi Province. The area of the Antaibao opencast coal mine is 375.12 km^2^, and it is the largest opencast coal mining area in China. This mining area has a typical temperate arid to semiarid continental monsoon climate and a fragile ecological environment with low coverage and high soil erosion. The annual mean temperature range is 5.4 °C–13.8 °C, and the total annual precipitation averages 426.7 mm, with 75–90% occurring during the rainy season (June–September). The average annual effective evaporation is approximately 2160 mm, almost five-fold greater than the amount of precipitation^40^. The study area was once primarily a landscape of forest and grassland; however, during the past 200 years, the primary vegetation has been damaged by long-term human disturbance and climate change, leading to chronic water and soil erosion.

The specific study areas were located in the South Dump, West Dump and Internal Dump of the Antaibao mine. The South Dump was reclaimed in 1992, and the West Dump and Internal Dump were reclaimed in 1995. The areas of the South Dump, West Dump and Internal Dump are 180.5 hm^2^, 280.16 hm^2^ and 264.4 hm^2^, respectively, and the peak elevations are 1465 m, 1520 m and 1500 m, respectively. The ecological environments of the three dumps have been effectively restored by a multi-level and multi-type forest–shrub–grass plant structure, which is presently composed of elms, black locusts, pines, willows and sea buckthorns.

There were three main revegetation models in the three dumps: Model A, black locust with a spacing of 1 m × 1 m; Model B, interline interplanting of Black locusts and Chinese pines, and the spacing of black locusts and Chinese pines was 1 m × 2 m and 5 m × 2 m, respectively; and Model C, interline interplanting of black locusts, elms and Ailanthus, and all the spacing of black locusts, elms and Ailanthus was 1 m × 1 m.

### Plot survey and sampling

**Figure 2:**
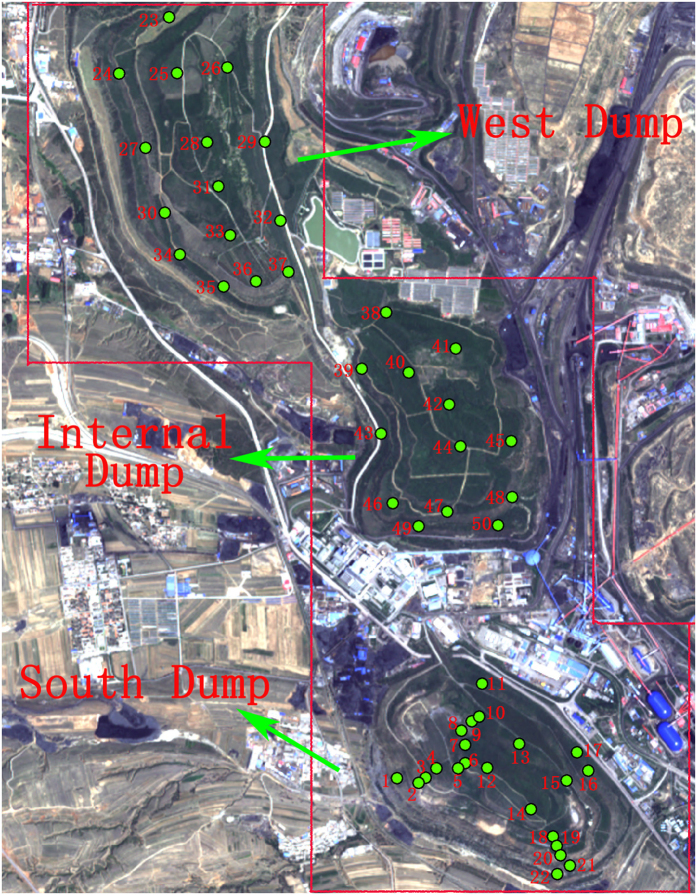
Layout of the sampling points at the Antaibao opencast coal-mine dumps, and it was processed using ArcGIS software based on SPOT satellite image obtained in 2014.

A plot survey was conducted at the three dumps of the Antaibao opencast coal mine in the summer from July 7–12 2014. Field measurements of the vegetation in the three dumps were performed in 50 sampling plots (Fig. 2). The number of sampling plots in the South Dump, West Dump and Internal Dump were 22, 15 and 13, respectively. South Dump included 2 belt transects with 1 to 22 sampling plots. The West Dump and Internal Dump included three belt transects, with 23 to 37 and 38 to 50 sampling plots, respectively. To include different topographic factors in the different sampling plots, the belt transects were laid in the northwest-southeast and northeast-southwest directions. Due to the limited topographic conditions, and taking into account that the selected plots should have the same or similar vegetation types, parts of the sampling plots were slightly off of the actual sampling sites.

One tree quadrat (10 m × 10 m) and three herb quadrats (1 m × 1 m) were set up in representative areas at each sampling plot. At each tree quadrat, the species, number, height, diameter at breast height (DBH), and canopy density were measured. The tree volume was calculated based on the number, height and DBH. At each herb quadrat, the herbage coverage was measured. In addition, all of the above-ground herbage was clipped, and any dead branches were removed. The remaining herbs were placed into a package. The above-ground biomass was measured in the laboratory. Moreover, each quadrat was labelled with a nail stake at the corner and located using the coordinates of the quadrat’s centre using GPS, and the longitude, latitude, elevation, slope, slope position, and slope aspect were recorded (Table 5).

Soil samples were collected at the 50 sampling plots in the three dumps after any plant litter was removed. At each sampling plot, three soil samples were randomly collected using the cutting ring from the surface layer (0–20 cm) for assessment of the soil physical properties, including the soil water content, soil bulk density, soil total porosity, and rock content. One mixed soil sample was also collected from the surface layer (0–20 cm) to determine the soil properties at each sampling plot, including the soil texture, total N, soil organic matter, available K and available P. All of the samples were serially numbered and stored in soil-bags for further analysis.

The soil water content, soil bulk density and soil total porosity were determined by the oven-drying method. The rock content was measured using the gravimetric method. The soil texture was divided into three classifications based on American systems: clay (<0.002 mm), silt (0.002–0.05 mm), and sand (0.05–2 mm). Soil particles of the soil samples were analysed using a Longbench Mastersizer 2000 laser particle-size analyser (Malvern Instruments, Malvern, England). The soil organic matter was determined by the thermal potassium dichromate oxidation colorimetric method^41^. The total N was determined by Kjeldahl digestion, distillation and titration^42^. The available P was determined by the molybdate colorimetric method after perchloric acid digestion and ascorbic acid reduction^43^. The available K was analysed using a flame atomic absorption spectrophotometer^44^.

The slope position and slope aspect were converted into a coded scale though the establishment of a membership function according to an empirical formula^45^. The slope aspect was classified as follows: a platform was given a value of 0, a sunny slope was given a value of 0.3, a half-sunny slope was given a value of 0.5, and a half-shaded slope was given a value of 1^46^,^47^. The slope position was classified as follows: a platform was given a value of 0.1, an upper slope was given a value of 0.4, a middle slope was given a value of 1, and a lower slope was given a value of 0.8^46^,^47^ (Table 6).

### Statistical analysis

Descriptive statistical analyses, including the calculations of mean, median, coefficient of variation (CV), maximum values, and minimum values, were performed to analyse the soil data. Tree volume was used to indicate the vegetation restoration in this study. All of the 14 soil and topographic factors were used to perform a one-way ANOVA and significance testing using SPSS 20.0 to explore the effects of environmental factors on tree volume. Differences were considered to be statistically significant at *P* < 0.05.

CANOCO 4.5 software was used to conduct multivariate analysis using detrended correspondence analysis (DCA), redundancy analysis (RDA) and canonical correspondence analysis (CCA) to study the effects of soil and topographic factors on vegetation restoration^48^. To determine whether a linear or unimodal-based numerical method should be used, DCA with detrending by segments was first conducted to analyse the vegetation data in order to evaluate the gradient length of the first axis. A linear model with RDA was the most useful when the gradient length of the first axis was less than 3 SD, whereas a unimodal model with CCA was suitable when it was greater than 4 SD. For intermediate lengths, both models can be useful^20^,^49^. The Monte Carlo permutation test was conducted to test the significance of the eigenvalues of the first canonical axis. Inter-set correlations from the ordination analysis were evaluated to assess the importance of various soil and topographic variables^50^. According to the obtained ordination plot, the correlation between the environmental factors (topographic and soil variables) and vegetation were analysed. Fourteen environmental variables (topographic and soil properties) were included in this analysis: slope, slope position, slope aspect, soil bulk density, soil total porosity, soil water content, rock content, total N, soil organic matter, available P, available K, clay content, silt content and sand content. The four vegetation variables included were tree volume, canopy density, above-ground biomass and herb coverage.

## Additional Information

**How to cite this article**: Wang, J. *et al.* Effects of soil and topographic factors on vegetation restoration in opencast coal mine dumps located in a loess area. *Sci. Rep.* **6**, 22058; doi: 10.1038/srep22058 (2016).

## Figures and Tables

**Table 1:** Descriptive statistical analysis of the soil data.

Soil factors	Number	Minimum	Maximum	Mean	Median	CV
Soil bulk density (g cm^−3^)	50	1.01	1.72	1.35	1.35	12%
Soil total porosity (%)	50	34.42	61.76	48.53	47.84	18%
Soil water content (g g^−1^)	50	3.44	8.74	6.45	6.44	13%
Rock content (%)	50	0	0.58	0.25	0.3	77%
Total N (%)	50	0.03	0.3	0.09	0	57%
Soil organic matter (%)	50	0.46	9.96	2.73	1.75	89%
Available P (mg kg^−1^)	50	2.42	11.63	4.91	4.29	44%
Available K (mg kg^−1^)	50	56	274	151.3	147.5	33%
Clay content (<0.002 mm, %)	50	0	25.1	8.39	1.53	109%
Silt content (0.002–0.05 mm, %)	50	12.1	51.17	28.63	28.22	34%
Sand content (0.005–2 mm, %)	50	42.3	85.49	62.98	64.39	16%

**Table 2:** Results of the one-way ANOVA and significance testing of the effect of soil and topographic factors on vegetation.

Influence factors	Single variable	F-ratio	P-value
Topographic factors	Slope	1.575	0.130
	Slope aspect	1.932	0.122
	Slope position	2.13	0.109
Soil factors	Soil bulk density	1.986	0.318
	Soil total porosity	1.986	0.318
	Soil water content	0.115	0.621
	Rock content	1.03	0.488
	Total N	8.741	0.108
	Soil organic matter	4.749	0.352
	Available P	1.56	0.364
	Available K	5.94	0.006**
	Clay(<0.002 mm)	0.446	0.997
	Silt(0.002–0.05 mm)	9.458	0.026*
	Sand(0.005–2 mm)	10.857	0.237

*significant difference at the 0.05 level; **significant difference at the 0.01 level.

**Table 3:** Ordination results of the RDA in the study area.

RDA axes	1	2	3	4	Total variance
Eigenvalues	0.333	0.025	0.013	0	1
Vegetation-environment correlations	0.636	0.492	0.432	0.295	
Cumulative percentage variance of vegetation data	33.3	35.8	37.1	37.1	
Cumulative percentage variance of vegetation-environment relationship	89.7	96.3	99.9	100	
Sum of all eigenvalues					1
Sum of all canonical eigenvalues					0.371
	**Trace**	**F-ratio**	**P-value**		
Test of significance of all canonical axes	0.675	5.6	0.016		

**Table 4:** Correlation analysis of influence factors in an opencast coal mine dump in a loess area.

Influence factors	Slope	Aspe	Posi	BD	SWC	BP	RC	TN	SoOM	AP	AK	Clay	Silt	Sand
Slop	1													
Aspe	0.1213	1												
Posi	0.5576**	0.1289	1											
BD	−0.1474	0.0069	−0.0004	1										
SWC	−0.002	0.1356	0.0956	0.1062	1									
BP	0.1082	−0.0104	−0.0834	−0.9606**	−0.0225	1								
RC	0.3779	0.0312	0.3347	−0.0472	0.0989	0.0853	1							
TN	0.1	0.2507	0.428*	−0.0326	−0.081	0.0563	0.2372	1						
SoOM	0.1047	0.0891	0.385	−0.0424	−0.0333	0.0609	0.226	0.8746**	1					
AP	−0.2126	0.1344	−0.1247	0.0605	0.1111	−0.0289	−0.019	−0.0098	−0.1645	1				
AK	−0.0945	0.1546	0.0005	0.207	0.1157	−0.1568	0.0619	0.3035	0.1909	−0.0454	1			
Clay	0.1622	−0.1087	−0.0757	−0.1817	0.0374	0.2151	0.1766	−0.5092*	−0.4684*	0.3111	−0.0878	1		
Silt	−0.2103	0.1279	−0.1196	0.1305	0.268	−0.1498	−0.1932	−0.0377	0.0718	−0.084	0.1395	−0.422*	1	
Sand	0.0548	−0.0243	0.1827	0.0391	−0.29	−0.0508	0.0255	0.4953*	0.3538	−0.2003	−0.0542	−0.4984*	−0.5756**	1

Abbreviations of soil and topographic variables: Posi, slope position; Aspe, slope aspect; SWC, soil water content; BD, soil bulk density; BP, soil total porosity; RC, rock content; SoOM, soil organic matter; TN, total N; AP, available P; and AK, available K.

**Table 5:** Basic information of the sampling plots.

Sample plot	Species	Revegetation year	Revegetation model	Elevation	Tree number	Average DBH (cm)	Average height (m)	Canopy density	Herbage coverage (%)
1	Black locust/Elm	1992	Model A	1375.01	19	17.9	3.9	0.15	35
2	Black locust	1992	Model A	1434.74	8	19.5	2.53	0.4	35
3	Black locust/Chinese pine/Poplar	1992	Model B	1408.68	32	7.8	1.82	0.05	60
4	Black locust/Elm	1992	Model A	1426.78	35	29.4	6.13	0.45	30
5	Black locust/Elm	1992	Model A	1450.05	35	35.3	6.01	0.18	10
6	Black locust/Elm	1992	Model A	1441.66	15	27	4.42	0.4	25
7	Black locust	1992	Model A	1444.1	27	36.2	8.17	0.6	5
8	Black locust/Elm	1992	Model A	1430.54	56	21.7	3.95	0.3	35
9	Black locust	1992	Model A	1402.74	11	26.8	4.6	0.2	25
10	Black locust	1992	Model A	1386.74	19	28.8	5.99	0.55	15
11	Black locust/Elm	1992	Model A	1329.16	32	25	4.82	0.52	35
12	Black locust/Elm/Chinese pine	1992	Model B	1454.21	35	27.5	5.1	0.6	5
13	Black locust/Elm/Ailanthus	1992	Model C	1375.55	28	27.1	7	0.6	20
14	Black locust/Elm	1992	Model A	1450.26	30	28.1	5.83	0.55	8
15	Black locust/Elm/Poplar	1992	Model A	1359.03	11	19	3.67	0.1	40
16	Black locust/Elm	1992	Model A	1344.35	9	24.89	8.49	0.2	40
17	Black locust/Chinese pine/Elm	1992	Model B	1351.54	28	37.6	7.53	0.65	20
18	Black locust	1992	Model A	1438.52	64	23.5	5.5	0.5	30
19	Black locust	1992	Model A	1431.64	75	17.1	6.88	0.4	10
20	Apricot/Chinese pine	1992	Model B	1391.25	160	16.9	3.82	0.85	5
21	Black locust/Elm	1992	Model A	1343.21	19	25.9	4.06	0.5	9
22	Black locust	1992	Model A	1362.8	13	39.9	7.67	0.25	70
23	Chinese pine	1995	Model B	1498.23	20	5.3	21.6	0.25	20
24	Elm/Chinese pine	1995	Model B	1484.46	21	5.16	30.63	0.4	25
25	Elm/Chinese pine	1995	Model B	1514.52	19	4.53	21.5	0.25	21
26	Elm	1995	Model C	1488.11	31	5.75	28	0.3	25
27	Elm	1995	Model C	1485.72	27	6.1	27.55	0.42	35
28	Chinese pine	1995	Model B	1519.56	61	4.34	22	0.4	2
29	Elm	1995	Model C	1457.43	6	5.72	41.4	0.25	28
30	Elm	1995	Model C	1456.52	23	7.33	32.2	0.49	18
31	Elm	1995	Model C	1499.13	40	4.63	28.31	0.62	9
32	Elm/Willow	1995	Model C	1470.44	9	5.28	34.51	0.28	30
33	Elm/Chinese pine	1995	Model B	1440.21	35	3.45	16.46	0.19	8
34	Chinese pine	1995	Model B	1436.4	22	6.18	30.26	0.23	30
35	Elm/Willow	1995	Model C	1426.25	12	5.98	37.2	0.21	21
36	Elm/Chinese pine	1995	Model B	1460.16	8	3.66	17.8	0.31	32
37	Elm	1995	Model C	1421.54	13	6.06	34.35	0.32	41
38	Elm/Willow	1995	Model C	1417.32	24	6.41	29.29	0.2	13
39	Elm	1995	Model C	1429.19	13	2.65	22.82	0.15	51
40	Elm/Chinese pine	1995	Model B	1441.71	52	3.56	24.93	0.42	6
41	Elm/Willow	1995	Model C	1425.86	17	5.73	28.53	0.31	12
42	Elm	1995	Model C	1433.46	9	5.92	37.8	0.15	32
43	Chinese pine	1995	Model B	1416.59	21	3.05	18.43	0.39	23
44	Elm/Chinese pine	1995	Model B	1372.35	14	4.73	34	0.31	36
45	Chinese pine	1995	Model B	1420.87	19	3.24	32.21	0.25	37
46	Elm	1995	Model C	1438.52	51	5	20.8	0.78	9
47	Chinese pine	1995	Model B	1420.34	10	3.86	29.55	0.31	32
48	Elm	1995	Model C	1423.59	90	6.42	21.8	0.3	2
49	Elm/Willow	1995	Model C	1401.69	14	4.98	37.36	0.15	5
50	Chinese pine	1995	Model B	1385.46	13	7.67	39.9	0.25	70

**Table 6:** Topography data of each sampling point in the study area.

Sample	Slope	Slope aspect	Slope position	Sample	Slope	Slope aspect	Slope position
1	0	0.5	0.1	26	40	1.0	0.1
2	0	0.5	0.1	27	34	0.5	0.1
3	41	0.5	1.0	28	0	0.3	0.1
4	34	0.5	1.0	29	19	0.5	0.4
5	0	0.5	0.4	30	32	0.3	0.4
6	0	0.3	0.1	31	0	0.3	0.1
7	41	0.5	1.0	32	0	0.3	0.1
8	23	0.5	0.4	33	43	0.5	0.4
9	36	0.5	0.4	34	39	0.3	0.4
10	0	0.5	0.1	35	0	0.3	0.1
11	19	0.3	0.4	36	43	0.3	0.1
12	19	1.0	0.4	37	0	0.3	1.0
13	0	1.0	0.1	38	41	1.0	1.0
14	0	0.3	0.1	39	35	1.0	0.4
15	42	0.5	0.4	40	0	0.0	0.1
16	5	1.0	0.1	41	0	0.5	0.1
17	12	0.8	1.0	42	41	0.3	0.4
18	28	0.3	0.4	43	0	0.3	0.1
19	0	1.0	0.1	44	0	0.5	0.1
20	0	0.3	0.1	45	25	0.5	0.4
21	45	0.3	1.0	46	0	0.5	0.1
22	40	0.5	0.8	47	42	0.3	0.4
23	0	0.5	0.1	48	23	0.5	0.4
24	0	0.5	0.1	49	9	0.5	0.4
25	23	0.5	0.4	50	0	0.5	0.1
